# Current surgical techniques for cleft lip-palate in Minas Gerais, Brazil

**DOI:** 10.1016/S1808-8694(15)30546-2

**Published:** 2015-10-19

**Authors:** Lívia Maris Ribeiro Paranaíba, Hudson de Almeida, Letízia Monteiro de Barros, Daniella Reis Barbosa Martellia, Julian Dias Orsi, Hercílio Martelli

**Affiliations:** 1 Master's degree student, Universidade Estadual de Campinas - Unicamp; 2 Medical resident, professor of plastic surgery, Universidade de Alfenas; 3 Doctorate, professor at the Universidade de Alfenas; 4 Master's degree student, professor at the Universidade Estadual de Montes Claros - Unimontes; 5 Master's degree, professor at the Universidade de Alfenas; 6 Doctorate, full professor at the Universidade Estadual de Montes Claros - Unimontes. Universidade Estadual de Montes Claros - Unimontes

**Keywords:** epidemiology, cleft lip, cleft palate, surgical technique

## Abstract

Cleft lip and palate (CL/P) are the most common congenital anomalies of the craniofacial region.

**Aim:**

to evaluate the surgical techniques used in CL/P treatment in a craniofacial deformities ward, in Minas Gerais.

**Materials and Methods:**

In this retrospective study, carried out between 2002 and 2007, we studied 109 individuals with non-syndromic CL/P submitted to treatment. The aspects analyzed (personal identification, classification of CL/P and surgical treatment performed) were obtained from patient charts, and then we built a database and ran statistical analyses through the SPSS 13.0 software. Followed by descriptive analysis of the surgical procedures depending on the type of CL/P found.

**Results:**

Among the 109 patients, 65.1% were males and 34.8% females. We found that 45% of patients had cleft lip and palate, 37.6% cleft lip only and 17.4% cleft palate only. The surgical techniques employed were predominantly those from Millard and Spina for cheiloplasty, McComb for rhinoplasty and, Veau and Van Langenbeeck for palatoplasty.

**Conclusions:**

This study is the first to address treatment procedures for individuals with CL/P in the state of Minas Gerais. For unilateral CL/P we predominantly used the association of McComb, Veau and Millard techniques, respectively, for rhinoplasty, palatoplasty and cheiloplasty, in 76.9% of the patients.

## INTRODUCTION

Neural tube defects and orofacial clefts are among the most common congenital anomalies. Although the etiology of these conditions may vary, genetic and environmental factors are both involved.^1^, ^2^ Orofacial clefts are present in 1:500 to 550 live births.^3^ Among the orofacial clefts, non-syndromic cleft lip and palate (CLP) comprise the most common alterations in the craniofacial area. In many parts of the world, the occurrence of CLP surpasses that of Down's syndrome.^4^

The incidence of CLP varies with geography, race, and social and economic status.^5^ Fogh-Andersen6 reported 1.5 cases of CLP for each 1,000 births in Denmark; the occurrence varied in other regions (1-2.69:1.000).^3^, ^4^ Recently, Martelli-Júnior et al.^7^ found 1.46 clefts for each 1,000 live births in the state of Minas Gerais, Brazil.

Recent studies on the etiology and pathogenesis of CLP have provided increasingly sophisticated clinical descriptions and revealed the genetics of this condition, particularly focusing on probable genes (such as the type 6 interferon regulating factor or IRF^6^) that may give rise to CLP.^8^, ^9^ Embryologically, clefts result from primary fusion defects of the craniofacial processes that form the primary and secondary palate in the first intrauterine trimester.^10^ These clefts may be classified anatomically, based on the incisive foramen, into four groups: pre-incisive foramen clefts or clefts lips (CL), post-incisive foramen clefts or cleft palates (CP), incisive transforamen clefts or cleft lip and palate (CLP), and rare facial clefts.^11^

Each cleft requires a multiprofessional approach to therapy, and surgery.^3^ Various surgical techniques and maneuvers have been developed to provide superior esthetic and functional repair to CLP patients since the initial work of Malgaine and Mirault in the 19th century.^12^ However, there is no consensus among professionals and specialized healthcare services about the ideal surgical approach.^13^ Many factors explains the lack of a standard approach, such as the difficulty in conducting longitudinal studies to demonstrate the effectiveness of each surgical technique for the various clinical forms of CLP.^13^

The aim of this study was to assess the most frequently used surgical techniques for the rehabilitation of CLP patients at a reference unit for craniofacial deformities in the state of Minas Gerais in Brazil.

## MATERIAL AND METHOD

A cross-sectional historical cohort study was undertaken to assess the surgical techniques used in the rehabilitation of CLP patients at a multiprofessional reference unit for craniofacial deformities located in the state of Minas Gerais, Brazil, from 2002 to 2007. The study included an analysis of 109 clinical files of patients with non-syndromic CLP diagnosed and fully treated at the reference unit, regardless of gender, age, race, place of birth or nationality. Patients with syndromic CLP, or that did not undergo the full rehabilitation that was proposed, were excluded from the sample.

Non-syndromic CLP were classified by using the incisive foramen^11^ as the anatomical reference landmark, as follows: (1) CL - complete or incomplete unilateral and bilateral pre-foramen clefts; (2) CLP - unilateral and bilateral transforamen, pre- and post-foramen clefts; (3) CP - all complete or incomplete post-foramen clefts; (4) Others - comprise all rare facial clefts. Surgical approaches were grouped by category of clinical procedures and anatomical regions as: cheiloplasty, rhinoplasty, and palatoplasty.

Clinical information, including personal identification, classification of CLP and surgical techniques applied in therapy, was gathered from the files of patients, to build a database for analysis using the statistical software SPSS, version 13.0 (Chicago, US). A descriptive analysis was made of the surgical techniques according to the type of CLP. This study was done in accordance with the law 196/68 of the National Health Board (Conselho Nacional de Saúde) under the Ministry of Health. The institutional review board of the university also approved this study (27/2005).

## RESULTS

The study population for this study of surgical techniques applied in the rehabilitation of non-syndromic CLP patients comprised 109 subjects aged from 2 to 55 years, of which 62 patients (56.9%) were white, 37 patients (33.9%) were brown, and 10 patients (9.2%) were black. The social and economic level was similar across the sample; patients were seen at a high complexity reference center of the Ministry of Health. There were 71 male subjects (65.1%) and 38 female subjects (34.8%).

Table 1 shows the prevalence of CLP in the sample (related with gender), and the percentage of each type within the general distribution of clefts. The study sample had no case of rare clefts. CLP was the most common type (45%), followed by CL (37.6%) and CP alone (17.4%).

**Table 1:** Type and prevalence of cleft lip and palate in the study sample.

		Gender (n)		General prevalence
FL/P type	n	Males	Females	(%)
Palatine fissure	19	11	8	17,4
Labial fissure	41	26	15	37,6
labio-palatine fissure	49	34	15	45,0
Total	109	71	38	100

Table 2 and 3 illustrate the surgical techniques applied in the rehabilitation of unilateral and bilateral CLP. The procedures used in the treatment of 76.9% of unilateral CLP were a combination of rhinoplasty, palatoplasty and cheiloplasty techniques, in particular the McComb, Veau and Millard methods. Cheiloplasty and palatoplasty were the most common procedures in the bilateral CLP group, in particular the Spina and Veau techniques.

**Table 2:** Surgical techniques for correcting unilateral cleft lip and palate.

		Males		Females	
Surgical technique	n	n	%	n	%
Cheiloplasty + Rhinoplasty	4	3	75	1	25
Cheiloplasty + Palatoplasty	5	3	60	2	40
Rhinoplasty + Palatoplasty	0				
Rhinoplasty + Palatoplasty + Cheiloplasty	30	23	76,6	7	23,3
Total	39	29	74,3	10	25,6

Cheiloplasty: Millard e Spina; Rhinoplasty: McComb e Reth; Palatoplasty: Veau e Furlow

**Table 3:** Surgical techniques for correcting bilateral cleft lip and palate.

		Males		Females	
Surgical technique	n	n	%	n	%
Cheiloplasty + Rhinoplasty	0				
Cheiloplasty + Palatoplasty	6	2	33,3	4	66,6
Rhinoplasty + Palatoplasty	0				
Rhinoplasty + Palatoplasty + Cheiloplasty	4	3	75	1	25
Total	10	5	50	5	50

Cheiloplasty: Millard e Spina; Rhinoplasty: McComb e Reth; Palatoplasty: Veau e Furlow

Table 4, Table 5 show the surgical procedures for correcting unilateral and bilateral CL. A combination of cheiloplasty and rhinoplasty techniques, particular the Millard and McComb approaches, were used in 64.8% of the unilateral CL group. Cheiloplasty alone, in particular Spina's technique, was used predominantly in bilateral CL. In CP alone cases (Table 6), 73.6% of 19 cases were rehabilitated using the Veau technique, followed in 21% of cases by the Van Langenbeeck technique.

**Table 4:** Surgical techniques for correcting unilateral cleft lip and palate.

		Males		Females	
Surgical technique	n	n	%	n	%
Cheiloplasty	13	8	61,5	5	38,4
Cheiloplasty + Rhinoplasty	24	15	62,5	9	37,5
Total	37	23	62,1	14	37,8

Cheiloplasty: Millard e Spina; Rhinoplasty: McComb e Reth

**Table 5:** Surgical techniques for correcting bilateral cleft lip and palate.

		Males		Females	
Surgical technique	n	n	%	n	%
Cheiloplasty	3	2	66,6	1	33,3
Cheiloplasty + Rhinoplasty	1	1	100	0	0
Total	4	3	75	1	25

Cheiloplasty: Millard e Spina; Rhinoplasty: McComb e Reth

**Table 6:** Surgical techniques for correcting cleft lip and palate.

		Males		Females	
Surgical technique	n	n	%	n	%
Palatoplasty					
Veau	14	7	50	7	50
Van Langenbeeck	4	3	75	1	25
Furlow	1	1	100	0	0
Total	19	11	57,9	8	42,1

## DISCUSSION

Several epidemiological studies have been undertaken to assess the distribution of CLP.^14^, ^15^ It is clear that the different types of clefts have distinct epidemiological distributions, and that incidences vary among population groups.^10^ CLP is more common in Asians, native Americans, Australian aborigines, and northern Europeans, while CP alone is found more often in Africans and their descendents.^9^ In this study, of 109 clefts, CLP was the most common type (45% of cases), followed by CL alone (37.6%) and CP (17.4%). A recent study of 126 Brazilian children with non-syndromic CLP showed that the prevalence of CLP in males was 2.57 times that of females, and that CLP occurred more often, followed by CL and CP alone.^16^ Our study concurs with these epidemiological findings; CLP predominated in males (1.86 times) compared to females. We also found that CLP occurred more often, compared to CL and CP alone.

Franco et al. (2003)^17^, assessed surgical protocols in CLP patients in Brazil and found that 75% of healthcare units had three of more specialists in the team, in particular plastic surgeons, dental surgeons and speech therapists. In 63% of these units, surgery was undertaken in less than five primary clinical cases per month. Thus, the experience of surgeons with cleft surgery is limited in 2/3 of these units; they do not consider the teams appropriate for the full rehabilitation of CLP patients.^17^ The present study was conducted in a reference healthcare unit^7^, ^16^ for the treatment of craniofacial deformity patients, especially CLP, and consists of a multidisciplinary team including plastic surgeons, dental surgeons, speech therapists, psychologists, nutritionists, otorhinolaryngologists, pediatricians, bucco-maxillo-facial prothesist, and physical therapists. Our unit also operates more than five primary cases per month, among other reasons because of the distance to other specialized reference centers.

A review of the surgical protocols in Brazilian specialized centers^17^ showed that the Millard cheiloplasty technique was preferred for unilateral cases, and that the Spina and Millard techniques were chosen for bilateral cases. Our results concur with these findings, both for unilateral and bilateral clefts. A combination of rhinoplasty, palatoplasty and cheiloplasty for unilateral CLP was observed, in particular the McComb, Veau and Millard techniques, respectively, in 76.9% of patients. Cheiloplasty and palatoplasty were applied more often in bilateral CLP patients, in particular the Spina and Veau techniques for each. Franco et al. (2003)^17^, found that palatoplasty techniques varied widely in the healthcare units they studies, depending on the clinical presentation. Our findings showed that the Veau and Van Langenbeeck techniques are more commonly applied for palatoplasty, which concurs with international reports.^18^, ^19^

A review of Brazilian surgical protocols^17^ revealed that from 1995 to 1999 the majority of CLP cases in both genders were rehabilitated in state of Sao Paulo (67%, 73%, 73%, 83% e 84%). At our unit, about 92% of treated patients came from the state of Minas Gerais, in particular the south and northern regions of this state. There were 62 white patients (56.9%), 37 brown patients (33.9%) and 10 black patients (9.2%).

Vieira (2008)^9^, published a recent study on the etiopathogeny of CLP in which he compared this biological event with a puzzle containing over 100 pieces, comprising from three to 14 genes and other risk factors. Although the participation of genes such as the IRF^6^, FGF (fibroblast growth factor), MSX1 (muscle segment homeobox) are fairly well known, as well as risk factors such as smoking in mothers, practical application of this knowledge remains limited. More effective therapy may become available in future after additional studies are done to further understand the effect of these agents in vitro and in animal models. Rehabilitation of CLP patients may take decades; decentralized healthcare units distributed symmetrical throughout the country could facilitate this treatment in Brazil.^17^ These units require multi- and interdisciplinary teams to optimize the treatment and rehabilitation. All patients in this study (n=109) were diagnosed and treated with a multi- interdisciplinary approach at our unit.

## CONCLUSION

This retrospective study showed that the Millard technique for unilateral cheiloplasty and the Spina and Millard techniques for bilateral cheiloplasty predominated in the treatment of non-syndromic CLP (n=109). A combination of rhinoplasty, palatoplasty and cheiloplasty techniques (McComb, Veau and Millard) was applied in the treatment of unilateral CLP in 76.9% of patients. Cheiloplasty and palatoplasty (Spina and Veau) was the most common combination for the treatment of bilateral CLP. The Veau and Van Langenbeeck procedures were the most commonly used palatoplasty techniques. Identifying and understanding the surgical protocols used in Brazilian specialized treatment units for the rehabilitation of CLP patients may facilitate comparisons and more effective programs for the therapy of these patients.
